# Association of quality of life and disease control with cigarette smoking in patients with severe asthma

**DOI:** 10.1590/1414-431X2021e11149

**Published:** 2022-01-05

**Authors:** V.C.H. dos Santos, M.A.F. Moreira, A.V. da Rosa, S.M. Sobragi, C.A.J. da Silva, P.T.R. Dalcin

**Affiliations:** 1 Programa de Pós-Graduação em Ciências Pneumológicas, Faculdade de Medicina, Universidade Federal do Rio Grande do Sul, Fundação Hospitalar Getúlio Vargas, Sapucaia do Sul, RS, Brasil; 2 Serviço de Pneumologia, Hospital das Clínicas de Porto Alegre, Porto Alegre, RS, Brasil; 3 Curso de Graduação, Faculdade de Fisioterapia, Universidade Federal do Rio Grande do Sul, Porto Alegre, RS, Brasil; 4 Programa de Pós-Graduação em Ciências Pneumológicas, Departamento de Medicina Interna, Faculdade de Medicina, Universidade Federal do Rio Grande do Sul, Serviço de Pneumologia, Hospital das Clínicas de Porto Alegre, Porto Alegre, RS, Brasil

**Keywords:** Adult, Asthma quality of life questionnaire, Cross-sectional study, Cigarette

## Abstract

More information is needed on asthma control and health-related quality of life (HRQoL) in smokers with severe asthma. The main study objective was to characterize the association of HRQoL and disease control with cigarette smoking in individuals with severe asthma. A secondary objective was to analyze subject characteristics according to asthma onset: asthma that developed before smoking initiation versus asthma that developed after smoking initiation. This cross-sectional study included subjects with severe asthma aged 18-65 years. HRQoL was assessed using the Asthma Quality of Life Questionnaire (AQLQ) and asthma control was assessed using the Asthma Control Test (ACT) and Global Initiative for Asthma (GINA) criteria. Of the 87 patients studied, 58 (66.7%) were classified as asthmatics who had never smoked and 29 (33.3%) as asthmatics with smoking exposure. The proportion of subjects with uncontrolled asthma was higher in the asthma and smoking group (GINA criteria: P=0.032 and ACT criteria: P=0.003. There were no between-group differences in overall AQLQ score (P=0.475) or AQLQ domain scores (P>0.05). Fifty-eight subjects (66.7%) were nonsmokers, 20 (23%) had asthma onset before smoking, and 9 (10.3%) had asthma onset after smoking. Asthma onset before smoking was associated with uncontrolled asthma (P=0.013). In subjects with severe asthma, smoking was associated with a higher rate of uncontrolled disease but not with HRQoL scores.

## Introduction

The aim of asthma treatment is to achieve disease control or maintain the current level of control and prevent future risks (exacerbations, instability of the disease, accelerated loss of lung function, and adverse effects of treatment) (1). Asthma control treatment is divided into steps 1 to 5, in which the dose of inhaled corticosteroids is progressively increased or other controller medications are added. Steps 4 and 5 represent more severe disease (2).

Severe asthma is asthma that is uncontrolled despite adherence to optimized high doses of inhaled corticosteroid/long-acting beta-agonist therapy and treatment of contributory factors. Currently, asthma severity is assessed retrospectively in terms of the level of treatment required to control symptoms and exacerbations. Asthma severity is assessed after the patient has been on controller treatment for several months and, if appropriate, treatment step-down has been attempted to determine the patient's minimum level of treatment (1,2). Although patients with severe asthma account for only 5-10% of asthma cases (2), they have higher morbimortality and account for a disproportionate use of healthcare resources compared with less severe groups (3).

Although smoking has declined considerably over the last 20 years in developed countries (4), cigarette smoking is still surprisingly common in asthma patients. The association between cigarette smoking and asthma can be harmful (5). In a Brazilian study (6), 7.8% of asthmatics were exposed to cigarette smoking.

Smoking is associated with worse levels of asthma control, more symptomatology, more frequent exacerbations, and higher mortality (7,8). Asthmatic smokers have more severe asthma symptoms, greater need for rescue medication, and worse health status indicators than individuals who have never smoked (9-11). Smoking is associated with greater lung function decline, increased health care costs, and lower therapeutic response to inhaled corticosteroids (12-14). Even ex-smoker asthmatics have higher asthma symptom scores and lower lung function than never smokers (5,15). Exposure to household air pollution and smoking is common in adults with asthma in Salvador, Brazil. Additionally, this dual exposure is associated with worse spirometric parameters, worse disease control, and greater asthma severity, indicating the harmful effect of this exposure (6).

It has been suggested that only asthma subjects with few symptoms and/or preserved lung function continue to smoke, and that subjects with more severe asthma refrain from smoking. This “healthy smoker” effect may explain the lack of association between cigarette smoke and asthma symptoms and the risk of developing asthma found in some studies (5).

Although smoking is known to worsen asthma control, there are few clinical studies on the association between severe asthma and smoking. There is a need for more information about asthma control and health-related quality of life (HRQoL) among adult smokers with severe asthma receiving tertiary care in southern Brazil.

Therefore, this study aimed to characterize the association of HRQoL and disease control with cigarette smoking in individuals with severe asthma. A secondary aim was to analyze patient characteristics according to asthma onset: asthma that developed before smoking initiation versus asthma that developed after smoking initiation.

## Material and Methods

This was a cross-sectional study approved by the Ethics and Research committee of the Hospital de Clínicas de Porto Alegre (HCPA), number 1.139.117. All study methods were carried out in accordance with international and national guidelines and regulations for clinical studies of humans (Declaration of Helsinki and Brazilian Governmental regulation - Plataforma Brasil). All patients signed an informed consent form.

Patients were recruited from the Asthma Outpatient Clinic of HCPA, Brazil. All patients who volunteered were sequentially included. The study included asthmatic patients aged between 18 and 65 years. The diagnosis was confirmed by compatible history of asthma and evidence of reversible airflow obstruction. This evidence was confirmed by spirometry showing airflow limitation, characterized by forced expiratory volume in the first second (FEV_1_) less than 80% of the predicted value and FEV_1_/forced vital capacity (FVC) ratio less than 75%; substantial improvement in airflow after inhalation of short-acting beta_2_-agonist bronchodilator (BD) (increase in FEV_1_ greater than 12% in relation to the pre-BD value and greater than 200 mL in absolute value); or increase in FEV_1_ greater than 20% and exceeding 250 mL spontaneously over time or after intervention with medication. All patients included in the study were being treated according to step 4 or 5 of the Global Initiative for Asthma (GINA) (16).

Asthmatic subjects exposed to smoking were included. Individuals who reported smoking in the past 30 days and had a smoking index higher than 5 pack-years were considered active smokers. Smokers in the cessation phase or ex-smokers were defined as individuals who had quit smoking during the last 30 days or more and had a smoking index greater than 5 pack-years. A control group of asthmatic nonsmokers included individuals who reported never having smoked and ex-smokers with a smoking index of less than 5 pack-years.

The following were excluded from the study: pregnant women, patients with other chronic lung diseases such as bronchiectasis, sequelae of pulmonary tuberculosis, diffuse lung fibrosis, lung neoplasm or neoplasm of other sites, human immunodeficiency syndrome, acquired immunodeficiency syndrome, or congenital immunodeficiency syndrome, psychiatric illness or incapacitating chronic neurological disease that could prevent the performance of the study procedures, and patients who did not complete the study evaluation tests.

The smoking index (pack-years) was calculated as follows: (number of cigarettes smoked per day/20) × the number of years the person had smoked.

Asthmatic patients were classified according to the temporal association between smoking initiation and asthma onset: asthma onset before smoking initiation, asthma onset after smoking initiation, and nonsmoking asthmatics.

HRQoL was assessed using Juniper's Asthma Quality of Life Questionnaire *(*AQLQ) (17,18). The AQLQ comprises four domains: symptoms, activity limitation, emotional function, and environmental exposure. Each domain is scored from 1 to 7; the score of 1 indicates maximal impairment and the score of 7 indicates no impairment.

The level of asthma control was assessed using the Asthma Control Test (ACT) (19) and the GINA table (16). The Charlson Comorbidity Index (20) was also used.

Spirometry was performed using a Jaeger v 4.31a spirometer (Jaeger, Germany). The following variables were recorded: FVC in liters and percentage of predicted value, FEV_1_ in liters and percentage of predicted value, and FEV_1_/FVC ratio in absolute value and percentage of predicted value. The carbon monoxide diffusing capacity (DL_CO_) was measured by a single sustained breath of a special gaseous mixture, using Master Screen Diffusion equipment (Jaeger). Pulmonary volumes were measured using the Master Screen Body-Plets (Jaeger). Lung function variables in nomograms were reported as a percentage of predicted values for gender, age, and height (21-23).

The six-minute walk test (6MWT) was performed according to the guidelines of the American Thoracic Society (24). Total distance walked in 6 min was recorded, and baseline and post-test peripheral oxygen saturation were measured using a pulse oximeter (NPB-40; Nellcor Puritan Bennett, USA).

A sample of the population underwent induced sputum examination to evaluate cellularity (25,26). We considered the sputum sample as eosinophilic if the percentage of eosinophils was ≥3% (27).

A blood count analysis was performed, and a total eosinophil count of 351 eosinophils/microliter or more was considered eosinophilia (26). The serum immunoglobin E (IgE) dose was analyzed and considered high if greater than 100 IU/mL, according to the reference value (28).

The skin prick test for allergy was conducted according to published protocols (29).

### Statistical analysis

All data were processed and analyzed using the Statistical Package for the Social Sciences, version 22.0 (SPSS Inc., USA). Quantitative variables are reported as means±SD or median and interquartile range. Categorical variables are reported using absolute and relative frequencies.

Pearson's chi-square test or Fisher's exact test were used to test for associations between categorical variables. To compare means, we used Student's *t*-test or one-way analysis of variance, supplemented by Tukey's test. In case of asymmetry, Mann-Whitney or Kruskal-Wallis test was used and supplemented by the Dunn's test.

A P-value ≤0.05 was considered statistically significant and all tests were two-tailed.

## Results

**Figure 1:**
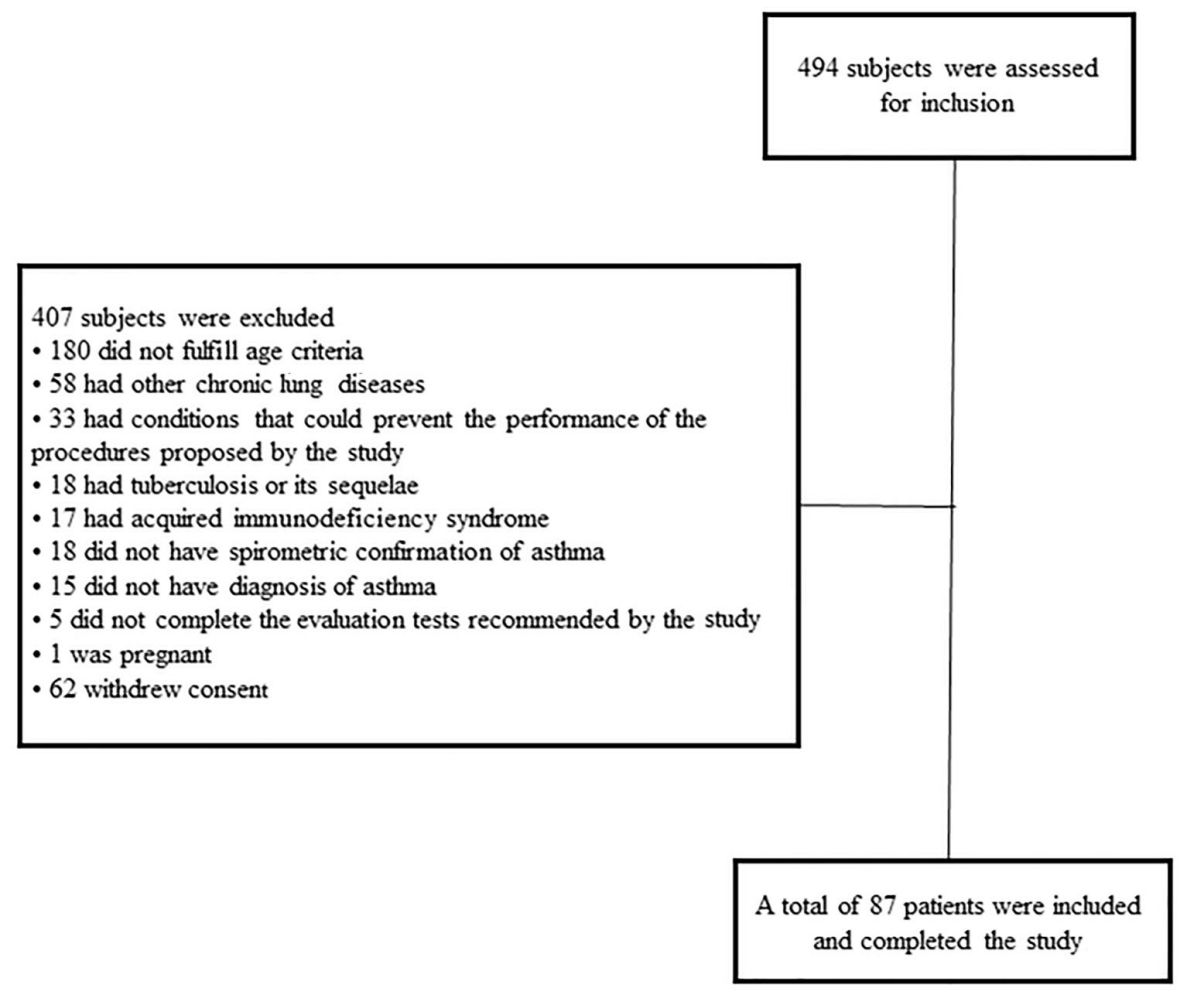
Flow diagram of study selection.

From October 2015 to June 2017, we assessed 494 adult subjects. Of these, 87 patients were included and completed the study (see Figure 1).

Table 1 shows the overall characteristics of the study sample. Of all subjects, 58 (66.7%) were never smokers, 22 (25.3%) were former smokers, and only 7 (8%) were current smokers.

**Table 1:** Characteristics of the patients.

Variables	Asthma with smoking exposure (n=29)	Asthma and never smokers (n=58)	P
Age (years), means±SD	55.8±8.5	42.4±13.3	<0.001
Gender, n (%)			0.293
Male	8 (27.6)	9 (15.5)	
Female	21 (72.4)	49 (84.5)	
Ethnicity, n (%)			0.528
White	24 (82.8)	43 (74.1)	
Non-white	5 (17.2)	15 (25.9)	
Marital status, n (%)			0.022
Single	5 (17.2)	25 (43.1)*	
Married	20 (69.0)*	20 (34.5)	
Separated/divorced/widowed	4 (13.7)	13 (22.4)	
Educational level, n (%)			0.06
≤8 years	13 (44.8)	16 (27.6)	
>8 years and <higher education	10 (34.5)	15 (25.9)	
≥higher education	6 (20.7)	27 (46.6)	
Work, n (%)			0.430
Yes, full shift	7 (24.1)	13 (22.4)	
Yes, half shift	4 (13.8)	15 (25.9)	
No	18 (62.1)	30 (51.7)	
Monthly income level, n (%)			0.683
≤US$ 231	8 (27.6)	19 (32.8)	
>US$ 231 to US$ 693	15 (51.7)	24 (41.4)	
>US$ 693	6 (20.7)	12 (20.7)	
Did not disclose	0 (0.0)	3 (5.2)	
Smoking status, n (%)			<0.001
Never smokers	0	58 (100)	
Current smokers	7 (24.1)	0	
Former smokers	22 (75.9)	0	
Smoking index (pack-years), median (IR)	25 (8.3-44.4)	0	-
Age of onset of symptoms (years), median (IR)	6 (2-23.5)	5.5 (1-30)	0.550
Age of diagnosis of asthma (years), median (IR)	16 (3.5-43)	11.5 (2-30)	0.056
BMI (kg/m^2^), means±SD	31.2±5.8	30.7±6.8	0.707
Charlson Comorbidity Index (points), median (IR)	3 (2-3)	1 (1-2)	<0.001

SD: standard deviation; n: number of cases; BMI: body mass index, IR: interquartile range (25th-75th percentiles). Pearson's chi-square test or Fisher's exact test for categorical variables; *t*-test for independent samples or Mann-Whitney test for continuous variables.

### Comparison between asthmatics with and without smoking exposure

The mean age was higher for asthmatics with smoking exposure (55.8±8.5 years) than for the never smokers group (42.4±13.3 years, P<0.001). The median Charlson score was higher in the asthma-smoking group (3 points) than in the asthma-never smoker group (1 point, P<0.001) (Table 1).

Table 2 compares HRQoL scores (AQLQ), asthma control, IgE levels, blood and sputum eosinophil counts, and skin prick test results between groups. There were no significant between-group differences for overall AQLQ score (P=0.475) or for AQLQ domain scores (symptoms, P=0.331; activity limitation, P=0.347; emotional function, P=0.589; environmental stimulus, P=0.449). The proportion of subjects with uncontrolled asthma was higher in the asthma-smoking group than in the asthma-never smoker group both for GINA criteria (respectively, 51.7 *vs* 48.3%; P=0.032) and ACT criteria <20 (respectively, 93.1 *vs* 63.8; P=0.003). The proportion of positive skin tests for allergies was lower in the asthma-smoking group than in the asthma-never smoker group (respectively, 34.5 *vs* 67.2%, P=0.007).

**Table 2:** Comparison of quality of life, asthma control, IgE, eosinophils, and skin test between groups.

Variables	Asthma with smoking exposure (n=29)	Asthma and never smokers (n=58)	P
AQLQ, means±SD	4.39±1.27	4.60±1.32	0.475
AQLQ Symptoms	4.65±1.20	4.94±1.36	0.331
AQLQ Activity Limitation	4.36±1.45	4.66±1.35	0.347
AQLQ Emotional Function	4.59±2.18	4.34±1.92	0.589
AQLQ Environmental Stimulus	3.43±1.78	3.75±1.87	0.449
Asthma control (GINA), n (%)			0.032
Controlled/partly controlled	8 (27.6)	21 (72.4)	
Uncontrolled	30 (51.7)	28 (48.3)	
Asthma control test, points - median (IR)	14 (12-16)	17 (11.8-21)	0.036
Asthma control test (<20 points; uncontrolled), n (%)	27 (93.1)	37 (63.8)	0.003
Serum IgE (IU/mL), median (IR)	74 (32-349)	154 (46-464)	0.169
IgE classification, n (%)			0.255
Normal (<100)	17 (58.6)	25 (43.1)	
High (>100)	12 (41.4)	33 (56.9)	
Serum eosinophils (eosinophils/μL), means±SD	0.29 (0.20-0.59)	0.24 (0.12-0.59)	0.400
Sputum eosinophils, n (%)			0.241
Unsuitable sample (saliva), n (%)	6 (25.0)	21 (43.8)	
<3%	13 (54.2)	17 (35.4)	
≥3%	5 (20.8)	10 (20.8)	
Skin test, n (%)			0.007
Positive	10 (34.5)	39 (67.2)	
Negative	19 (65.5)	19 (32.8)	

n: number of cases; AQLQ: Asthma Quality of Life Questionnaire; GINA: Global Initiative for Asthma; IgE: immunoglobulin E; IR: interquartile range (25th-75th percentiles). Pearson's chi-square test or Fisher's exact test for categorical variables; *t*-test for independent samples or Mann-Whitney test for continuous variables.

Table 3 compares pulmonary function and 6MWT results. At the time of diagnosis, mean values of pre-BD and post-BD FEV_1_ (% of predicted value) were lower for the asthma-smoker group (respectively, 58.5±16.7% and 71.8±16.6% of predicted value) than for the asthma-never smoker group (respectively, 69.9±18.1% and 82.5±18.6%; P=0.006 and P=0.011). At study inclusion, mean values of pre-BD FVC, pre-BD FEV_1_, pre-BD FEV_1_/FVC, and DLco were significantly lower for the asthma-smoking group (respectively, 73.6±16.1, 59.5±18.7, 65.4±11.7, and 67.9±22.6% of predicted value) than for the asthma-never smoker group (respectively, 83.5±16.0, P=0.008; 73.5±18.2, P=0.001; 72.5±9.4, P=0.003; and 80.3±15.5, P=0.013). The asthma-smoking group had significantly higher functional residual capacity values (136.7±32.2% of predicted value) than the asthma-never smoker group (121.7±27.6% of predicted value; P=0.027).

**Table 3:** Pulmonary function and 6-minute walk test.

Variables	Asthma with smoking exposure (n=29)	Asthma and never smokers (n=58)	P
Pulmonary function, mean±SD			
At diagnosis			
Pre-BD FVC (% predicted)	74.5±16.8	85.2±18.4	0.011
Pre-BD FEV_1_ (% predicted)	58.5±16.7	69.9±18.1	0.006
Pre-BD FEV_1_/FVC (%)	62.8±11.3	66.5±12.7	0.193
Post-BD FVC (% predicted)	88.0±14.2	94.5±19.8	0.124
Post-BD FEV_1_ (% predicted)	71.8±16.6	82.5±18.6	0.011
Post-BD FEV_1_/FVC (%)	66.9±13.7	74.4±12.3	0.012
At inclusion in the study			
Pre-BD FVC (% predicted)	73.6±16.1	83.5±16.0	0.008
Pre-BD FEV_1_ (% predicted)	59.5±18.7	73.5±18.2	0.001
Pre-BD FEV_1_/FVC (%)	65.4±11.7	72.5±9.4	0.003
Post-BD FVC (% predicted)	80.1±14.2	86.5±16.9	0.080
Post-BD FEV_1_ (% predicted)	67.0±17.8	79.3±18.2	0.004
Post-BD FEV_1_/FVC (%)	69.4±13.2	78.4±11.5	0.002
DLCOc SB (% predicted)	67.9±22.6	80.3±15.5	0.013
TLC (% predicted)	112.4±13.4	108.9±20.1	0.406
FRC (% predicted)	136.7±32.2	121.7±27.6	0.027
RV (% predicted)	177.8±50.5	165.2±47.1	0.252
Distance walked in 6-MWT (m), mean±SD	436.3±75.7	459.4±74.6	0.181

n: number of cases; BD: short-acting β_2_-agonist bronchodilator; FVC: forced vital capacity; FEV_1_: forced expiratory volume in the first second; DLCOc SB: diffusing capacity for carbon monoxide adjusted for hemoglobin; TLC: total lung capacity, FRC: functional residual capacity; RV: residual volume; 6MWT: 6-min walk test. *t*-test for independent samples or Mann-Whitney test.

### Comparison of asthma onset among groups

Table 4 compares the “asthma onset before smoking,” “asthma onset after smoking,” and “nonsmoking asthmatic” groups. There were 58 (66.7%) subjects in the nonsmoking group, 20 subjects (23%) in the group with asthma onset before smoking, and 9 subjects (10.3%) in the group with asthma onset after smoking. The nonsmoking asthmatic group was younger than the other two groups (respectively, 42.4±13.3, 58.6±5.1, 54.6±9.5; P<0.001). The median Charlson comorbidity indexes were significantly higher in the groups with asthma onset before smoking and asthma onset after smoking than in the nonsmoking asthmatic group (P<0.001). The proportion of subjects with uncontrolled asthma was higher in the group with asthma onset before smoking (80% GINA classification, P=0.048; 95% for ACT score, P=0.013). Pre-BD values of FVC, FEV_1_, FEV_1_/FVC, and DLco were higher for the group with asthma onset before smoking.

**Table 4:** Characteristics of patients according to the onset of asthma: asthma developed before starting cigarette smoking versus asthma developed after initiating cigarette smoking.

Variables	Asthma onset before smoking (n=20)	Asthma onset after smoking (n=9)	Nonsmoking asthmatics (n=58)	P
Age (years), mean±SD	54.6±9.5^b^	58.6±5.1^b^	42.4±13.3^a^	<0.001
Gender, n (%)				0.017
Male	8 (40.0)*	0 (0.0)	9 (15.5)	
Female	12 (60.0)*	9 (100)	49 (84.5)	
Age at diagnosis of asthma (years), median (IR)	6.5 (2.3-35.3)	44 (23.5-49.5)	11.5 (2-30)	0.770
BMI (kg/m^2^), mean±SD	31.7±4.5	30.2±8.3	30.7±6.8	0.789
Charlson Comorbidity Index, median (IR)	3 (2-3)^b^	3 (2-4)^b^	1 (1-2)^a^	<0.001
Asthma control (GINA), n (%)				0.048
Controlled/partially controlled	4 (20.0)*	4 (44.4)	30 (51.7)*	
Uncontrolled	16 (80.0)*	5 (55.6)	28 (48.3)*	
Asthma control test, points-median (IR)	14 (12-17.5)	14 (11.5-15)	17 (11.8-21)	0.110
Asthma control test, <20 points (well controlled), n (%)	19 (95.0)*	8 (88.9)	37 (63.8)*	0.013
Quality of life, mean±SD				
AQLQ	4.62±1.03	3.88±1.64	4.60±1.32	0.285
AQLQ Symptoms	4.80±0.95	4.33±1.66	4.94±1.36	0.426
AQLQ Activity Limitation	4.67±1.11	3.67±1.92	4.66±1.35	0.128
AQLQ Emotional Function	4.97±2.26	3.73±1.82	4.34±1.92	0.267
AQLQ Environmental Stimulus	3.51±1.73	3.25±1.99	3.75±1.87	0.706
Pulmonary function, mean±SD				
Pre-BD FVC (% predicted)	74.2±16.9^a^	72.4±14.9^ab^	83.5±16.0^b^	0.030
Pre-BD FEV_1_ (% predicted)	60.1±19.0^a^	58.1±19.2^ab^	73.5±18.2^b^	0.005
Pre-BD FEV_1_/ FVC (%)	65.9±12.4^a^	64.1±10.6^ab^	72.5±9.4^b^	0.011
Post-BD FVC (% predicted)	82.0±15.0	75.8±11.7	86.5±16.9	0.136
Post-BD FEV_1_ (% predicted)	69.4±18.4^ab^	61.7±16.3^a^	79.3±18.2^b^	0.009
Post-BD FEV_1_/ FVC (%)	70.4±12.5^a^	67.2±15.2^a^	78.4±11.5^b^	0.005
DLCOc SB (% predicted)	69.9±23.5^ab^	63.6±21.1^a^	80.3±15.5^b^	0.012
TLC (% predicted)	112.2±11.6	113.0±17.6	108.9±20.1	0.705
FRC (%)	136.0±32.2	138.3±34.3	121.7±27.6	0.085
RV (% predicted)	182.3±51.2	167.8±50.4	165.2±47.1	0.394
Distance walked in 6MWT (m), mean±SD	448.3±84.3	409.7±44.8	459.4±74.6	0.180
Eosinophilia (cells/µL), mean ±SD	252±186	267±270	331±306	0.506
Sputum eosinophils, n (%)				0.458
Unsuitable sample (saliva), n (%)	5 (27.8)	10 (55.6)	3 (16.7)	
<3%	1 (16.7)	3 (50)	2 (33.3)	
>3%	21 (43.8)	17(35.4)	10 (20.8)	
IgE (IU/mL), median (IR)	117 (65.7-441.7)	56 (34.1-171.1)	192 (79.8-490)	0.139

n: number of cases; BMI: body mass index; GINA: Global Initiative for Asthma; ACT: Asthma Control Test; AQLQ: Asthma Quality of Life Questionnaire; BD: short-acting β_2_-agonist bronchodilator; FVC: forced vital capacity; FEV_1_: forced expiratory volume in the first second; TLC: total lung capacity; FRC: functional residual capacity; RV: residual volume; 6MWT: 6-min walk test; IgE: immunoglobulin E; IR: interquartile range (25th-75th percentiles). Pearson's chi-square test or Fisher's exact test for categorical variables; *adjusted residual >1.96 or < -1.96 (implies significantly different percentages). Analysis of variance for continuous variables and Tukey *post hoc* test or Kruskal-Wallis tests and Dunn's *post hoc* test: different letters indicate significantly different means.

### *Post hoc* analysis

We performed a comparison between never smokers, current smokers, and former smokers. There was a significant age difference at study inclusion (41.6±18.9 years, 45.0±11.4 years, and 56.9±7.5 years, respectively; P<0.0001). There was also a significant difference in ACT scores (17.1±5.6, 11.5±3.7, and 15.4±4.2, respectively; P=0.039) and no differences in gender, lung function at study inclusion, and HRQoL scores.

## Discussion

In this cross-sectional study of patients with severe asthma treated at a large, university-affiliated tertiary care hospital in southern Brazil, we observed a higher rate of uncontrolled asthma in asthmatics who smoked than in non-smoker asthmatics in terms of both GINA criteria and ACT criteria. There were no significant between-group differences in overall AQLQ scores or AQLQ domain scores. Additionally, 66.7% of subjects were classified as non-smokers, 23% as having asthma onset before smoking, and 10.3% as having asthma onset after smoking. The proportion of subjects with uncontrolled asthma was higher in the group with asthma onset before smoking. There was no association between group and AQLQ scores.

It is worth noting that in our sample, only 7 (8%) subjects were current smokers. This low proportion of current smokers can be explained by the intensive educational efforts toward smoking cessation in the severe asthma outpatient clinic. This finding may also partly reflect the healthy smoker effect, in which only individuals with few asthma symptoms and/or preserved lung function continue to smoke, and subjects with more severe asthma refrain from smoking (5). Because of this limitation, data for current and former smokers were combined for analysis to form the group with asthma associated with smoking.

Althuis et al. (10) examined the association between cigarette smoking and asthma symptom severity in 225 asthmatic patients aged 20-54 years. Similar to our findings, they showed that cigarette smoking was associated with a higher frequency of asthma symptoms. However, in adjusted multivariate analyses, the relationship between smoking and higher overall asthma symptoms was of borderline significance (P=0.06).

Siroux et al. (11) analyzed 200 adult asthmatic subjects, 265 non-asthmatic controls, and 586 relatives of asthmatics (147 with asthma). The inclusion criteria classified asthmatics as mild, moderate, or severe. They concluded that active smoking is not a risk factor for asthma in adulthood, but that smoking increases asthma severity. Current smokers compared with never and ex-smokers had more asthma symptoms, more frequent asthma attacks, and higher asthma severity scores.

Dalcin et al. (30) investigated asthma control in 275 patients aged 11 years or older. In contrast to the present findings, there was no association with smoking status, but the study included patients with a wide age range and different severity classification (52.4% were classified as severely asthmatic, 33.8% as moderate, and 17.5% as mild).

Our finding that the groups with asthma associated with smoking and with asthma not associated with smoking did not differ on AQLQ overall or domain scores was unexpected. There are several explanations for this finding. First, patients with more severe forms of asthma may have worse HRQoL, regardless of smoking status (31). Second, this finding may reflect, at least partly, the healthy smoker effect (5). Third, the sample size may have been too small for the magnitude of the differences in AQLQ scores.

HRQoL was an important outcome, because the effect of asthma on activities of daily life is very important for patients, although it remains unclear which tool is the most suitable for assessing HRQoL. There are eleven instruments for adults that measure the impact effect of asthma on quality of life, but none is qualified as core instruments because they predominantly measure indicators of asthma control (symptoms and/or functional status), fail to provide a distinct, reliable score for measuring all key dimensions of the intended construct, and/or lack adequate psychometric data (32).

Boulet et al. (33) studied corticosteroid-naive asthma patients (no treatment for asthma used except for short-acting beta_2_-agonists on demand) and compared the clinical features of 22 smoking asthma patients and 27 non-smoking asthma patients. Similar to our own findings, the mean HRQoL global score was similar in smokers and nonsmokers. There were no differences in symptoms, activities, emotions, or environment. In contrast to the present findings, other studies (11,31,34) have demonstrated substantial associations between smoking and HRQoL in asthma subjects. However, most of the patients in these studies had mild or moderate asthma.

In the present study, the proportion of positive skin tests for allergies was significantly lower in the group with asthma associated with smoking. Skin tests are used in addition to a directed history and physical exam to exclude or confirm IgE-mediated diseases such as allergic rhinitis and asthma and anaphylaxis to aeroallergens, foods, insect venoms, and certain drugs.

Many chronic conditions and their treatments affect the response to asthma treatments or increase the difficulty of achieving asthma control. It has been demonstrated that comorbidities can adversely affect the long-term course of severe asthma (35). In this study, we found that non-smoker asthmatics had lower Charlson comorbidity scores than smokers.

Compared with subjects with asthma not associated with smoking, asthma associated with smoking had lower mean expiratory flow and lung diffusion capacity, and higher functional residual capacity. Several other studies have found worse lung function values in asthmatic smokers (13,14,33,36,37).^.^ Most subjects in our group with asthma associated with smoking met the criteria for asthma-chronic obstructive pulmonary disease syndrome (38).

There is a lack of studies that sufficiently characterize possible asthma phenotypes associated with asthma onset: asthma that developed before smoking initiation versus asthma that developed after smoking initiation. In this study, 23% of subjects had asthma onset before smoking and only 10.3% had asthma onset after smoking. The proportion of uncontrolled asthma was higher in subjects who had asthma onset before smoking. Lung function was worse in those who had asthma onset before smoking. Raherison et al. (36) examined asthma phenotypes according to the timing of smoking onset in young adults. Forty-six subjects (48%) were nonsmokers and 50 (52%) were current or former smokers; 39 had had their first asthma attack before starting smoking, and 11 had started smoking before having their first asthma attack. Asthma that developed before smoking initiation and asthma without smoking were both strongly related to nasal allergy, parental asthma, and atopy. Only a lower FEV_1_ level was substantially associated with asthma after smoking initiation. It is possible that, at least in some subjects, smoking requires an additional comorbid condition to influence asthma development or clinical expression (39).

The present study has some potential limitations. First, this was a cross-sectional study, so it was not possible to establish a temporal link between smoking in asthma subjects and quality of life. Second, the study was conducted in a single center and the sample was small. Third, there was an age difference between the groups with asthma associated with smoking and asthma not associated with smoking. This difference may reflect differences in phenotypes and in disease and severity progression. The effect of asthma on HRQoL is likely to differ according to several patient factors, including age. Fourth, in the current study we did not perform a direct correlation or association analysis between quality of life and asthma control for smoking history.

In conclusion, this cross-sectional study of patients with severe asthma identified a higher rate of uncontrolled asthma in subjects with asthma associated with smoking than in those with asthma not associated with smoking. However, there were no between-group differences in HRQoL scores. In addition, 66.7% of subjects were classified as nonsmoking, 23% as having asthma onset before smoking, and only 10.3% as having asthma onset after smoking. For better clarification of the association of HRQoL and disease control with cigarette smoking in severe asthma, further longitudinal studies with a large sample size are needed.

## References

[1] Pizzichini MMM, de Carvalho-Pinto RM, Cançado JED, Rubin AS, Neto AC, Cardoso AP (2020). 2020 Brazilian Thoracic Association recommendations for the management of asthma. J Bras Pneumol.

[2] GINA (Global Initiative for Asthma) Global strategy for asthma management and prevention (2021 update) 2021. https://ginasthma.org/wp-content/uploads/2021/05/GINA-Main-Report-2021-V2-WMS.pdf.

[3] Serra-Batlles J, Plaza V, Morejón E, Comella A, Brugués J (1998). Costs of asthma according to the degree of severity. Eur Respir J.

[4] WHO (World Health Organization) (2021). Data and Statistics 2021.

[5] Cerveri I, Cazzoletti L, Corsico AG, Marcon A, Niniano R, Grosso A (2012). The impact of cigarette smoking on asthma: a population-based international cohort study. Int Arch Allergy Immunol.

[6] Fernandes AGO, de Souza-Machado C, Pinheiro GP, de Oliva ST, Mota RCL, De Lima VB (2018). Dual exposure to smoking and household air pollution is associated with an increased risk of severe asthma in adults in Brazil. Clin Transl Allergy.

[7] Mcleish AC, Zvolensky MJ (2010). Asthma and cigarette smoking: a review of the empirical literature. J Asthma.

[8] Harmsen L, Gottlieb V, Makowska Rasmussen L, Backer V (2010). Asthma patients who smoke have signs of chronic airflow limitation before age 45. J Asthma.

[9] Thomson NC, Chaudhuri R, Livingston E (2004). Asthma and cigarette smoking. Eur Respir J.

[B10] Althuis MD, Sexton M, Prybylski D (1999). Cigarette smoking and asthma symptom severity among adult asthmatics. J Asthma.

[11] Siroux V, Pin I, Oryszczyn MP, Le Moual N, Kauffmann F (2000). Relationships of active smoking to asthma and asthma severity in the EGEA study. Eur Respir J.

[12] Gallefoss F, Bakke PS (2003). Does smoking affect the outcome of patient education and self-management in asthmatics?. Patient Educ Couns.

[B13] Thomson NC, Shepherd M, Spears M, Chaudhuri R (2006). Corticosteroid insensitivity in smokers with asthma: clinical evidence, mechanisms, and management. Treat Respir Med.

[14] Chalmers GW, Macleod KJ, Little SA, Thomson LJ, McSharry CP, Thomson NC (2002). Influence of cigarette smoking on inhaled corticosteroid treatment in mild asthma. Thorax.

[15] Kim SY, Sim S, Choi HG (2018). Active and passive smoking impacts on asthma with quantitative and temporal relations: a Korean Community Health Survey. Sci Rep.

[16] GINA (Global Initiative for Asthma) (2018). 2019 GINA Report: Global Strategy for Asthma Management and Prevention 2019.

[17] da Silva LMC, da Silva LCC (2007). Validation of asthma quality of life questionnaire (Juniper) to Brazilian Portuguese [in Portuguese]. Rev AMRIGS.

[18] Juniper EF, Guyatt GH, Epstein RS, Ferrie PJ, Jaeschke R, Hiller TK (1992). Evaluation of impairment of health related quality of life in asthma: development of a questionnaire for use in clinical trials. Thorax.

[19] Roxo JPF, Ponte EV, Ramos DCB, Pimentel L, D’Oliveira A, Cruz AA (2010). Portuguese-language version of the asthma control test: validation for use in Brazil [in Portuguese]. J Bras Pneumol.

[20] Charlson ME, Pompei P, Ales KL, MacKenzie CR (1987). A new method of classifying prognostic comorbidity in longitudinal studies: development and validation. J Chronic Dis.

[21] Prata TA, Mancuzo E, de Castro Pereira CA, de Miranda SS, Sadigursky LV, Hirotsu C (2018). Spirometry reference values for black adults in Brazil. J Bras Pneumol.

[B22] Crapo RO, Morris AH, Clayton PD, Nixon CR (1982). Lung volumes in healthy nonsmoking adults. Bull Eur Physiopathol Respir.

[23] Park JO, Choi IS, Park KO (1986). Normal predicted values of single-breath diffusing capacity of the lung in healthy nonsmoking adults. Korean J Intern Med.

[24] Crapo RO, Casaburi R, Coates AL, Enright PL, MacIntyre NR, McKay RT (2002). ATS statement: guidelines for the six-minute walk test. Am J Respir Crit Care Med.

[25] Gonçalves J, Pizzichini E, Pizzichini MMM, Steidle LJM, Rocha CC, Ferreira SC (2014). Reliability of a rapid hematology stain for sputum cytology. J Bras Pneumol.

[26] Pavord ID, Pizzichini MMM, Pizzichini E, Hargreave FE (1997). The use of induced sputum to investigate airway inflammation. Thorax.

[27] Chakir J, Loubaki L, Laviolette M, Milot J, Biardel S, Jayaram L (2010). Monitoring sputum eosinophils in mucosal inflammation and remodelling: a pilot study. Eur Respir J.

[28] de Azavedo J, Blake S, Dowling FB, Greally J (1978). Serum immunoglobulin E levels in normal healthy adults. Ir J Med Sci.

[29] Wood RA, Phipatanakul W, Hamilton RG, Eggleston PA (1999). A comparison of skin prick tests, intradermal skin tests, and RASTs in the diagnosis of cat allergy. J Allergy Clin Immunol.

[30] Dalcin PTR, Menegotto DM, Zanonato A, Franciscatto L, Soliman F, Figueiredo M (2009). Factors associated with uncontrolled asthma in Porto Alegre, Brazil. Braz J Med Biol Res.

[31] Wilson SR, Rand CS, Cabana MD, Foggs MB, Halterman JS, Olson L (2012). Asthma outcomes: quality of life. J Allergy Clin Immunol.

[32] Gonzalez-Barcala FJ, de La Fuente-Cid R, Tafalla M, Nuevo J, Caamaão-Isorna F (2012). Factors associated with health-related quality of life in adults with asthma. A cross-sectional study. Multidiscip Respir Med.

[33] Boulet LP, Lemiàre C, Archambault F, Carrier G, Descary MC, Deschesnes F (2006). Smoking and asthma: clinical and radiologic features, lung function, and airway inflammation. Chest.

[34] Tan NC, Ngoh SHA, Teo SSH, Swah TS, Chen Z, Tai BC (2012). Impact of cigarette smoking on symptoms and quality of life of adults with asthma managed in public primary care clinics in Singapore: a questionnaire study. Prim Care Respir J.

[35] Chen W, Marra CA, Lynd LD, Fitzgerald JM, Zafari Z, Sadatsafavi M (2016). The natural history of severe asthma and influences of early risk factors: a population-based cohort study. Thorax.

[36] Raherison C, Baldi I, Tunon-De-Lara JM, Taytard A, Annesi-Maesano I (2003). Asthma phenotypes according to the timing of smoking onset in young adults. Int J Tuberc Lung Dis.

[37] Tomlinson JEMM, McMahon AD, Chaudhuri R, Thompson JM, Wood SF, Thomson NC (2005). Efficacy of low and high dose inhaled corticosteroid in smokers *versus* non-smokers with mild asthma. Thorax.

[38] Postma DS, Rabe KF (2015). The asthma-COPD overlap syndrome. N Engl J Med.

[39] Boulet LP (2009). Influence of comorbid conditions on asthma. Eur Respir J.

